# Megacolon

**Published:** 1912-09

**Authors:** 


MEGACOLON.—D. Gerota, *Bull. of Soc. of Med. Sci. of Bucharest,* 1909-10, page 24. Civil engineer, intermittent obstipation since infancy, 10 days duration in 1908, section contrary to advice of consultants as last resort. Pelvic colon twisted twice on its axis, enormously distended. On account of desperate condition of patient, operation limited to untwisting. Temporary recovery. 10 months later, radical operation early in subsequent attack of obstipation. 85 c. m. of pelvic colon removed, 30-50 c. m. in circumference, 1, 1½ and 2 c. m. in thickness. Good recovery. Almost identical case, with two years between untwisting and radical operations, reported in discussion by Poenaro-Caplesco.

